# The multiple combination of Paclitaxel, Ramucirumab and Elacridar reverses the paclitaxel-mediated resistance in gastric cancer cell lines

**DOI:** 10.3389/fonc.2023.1129832

**Published:** 2023-02-16

**Authors:** Annalisa Schirizzi, Marialessandra Contino, Livianna Carrieri, Chiara Riganti, Giampiero De Leonardis, Maria Principia Scavo, Maria Grazia Perrone, Morena Miciaccia, Joanna Kopecka, Maria Grazia Refolo, Claudio Lotesoriere, Nicoletta Depalo, Federica Rizzi, Gianluigi Giannelli, Caterina Messa, Rosalba D’Alessandro

**Affiliations:** ^1^ Laboratory of Experimental Oncology, National Institute of Gastroenterology - Istituto di Ricovero e Cura a Carattere Scientifico (IRCCS) “Saverio de Bellis”, Castellana Grotte, BA, Italy; ^2^ Department of Pharmacy – Drug Sciences, University of Bari “A. Moro”, Bari, Italy; ^3^ Laboratory of Personalized Medicine, National Institute of Gastroenterology - IRCCS “Saverio de Bellis”, Castellana Grotte, BA, Italy; ^4^ Department of Oncology, University of Turin, Torino, Italy; ^5^ Medical Oncology Unit, National Institute of Gastroenterology - IRCCS “Saverio de Bellis”, Castellana Grotte, BA, Italy; ^6^ Institute for Chemical-Physical Processes, Italian National Research Council Istituto per i Processi Chimico Fisici (IPCF) - Consiglio Nazionale delle Ricerche (CNR) Sede distaccata o Secondaria (SS) Bari, Bari, Italy; ^7^ Department of Chemistry, University of Bari “A. Moro”, Bari, Italy; ^8^ Scientific Direction, National Institute of Gastroenterology - IRCCS “Saverio de Bellis”, Castellana Grotte, BA, Italy

**Keywords:** Paclitaxel-resistance, VEGF signaling, P-glycoprotein, combined treatment, exosomes

## Abstract

**Introduction:**

Paclitaxel (PTX) interferes with microtubule architecture by binding to β-tubulin, thereby blocking progression at the G2/M phase and inducing apoptosis. This study aimed to investigate molecular processes underlying PTX-mediated resistance in gastric cancer (GC) cells.

**Methods:**

PTX-mediated resistance involves many processes, and in this work some of the factors involved in the resistance mechanism were identified by comparing two GC lines with PTX induced resistance to their sensitive counterparts.

**Results:**

Thus, the key feature of PTX-resistant cells was the overexpression of pro-angiogenic factors such as VEGFA, VEGFC, and Ang2, known to support tumor cell growth. A second relevant change detected in PTX-resistant lines was the elevated level of TUBβIII, a tubulin isoform that opposes microtubule stabilization. A third identified factor contributing to PTX-resistance was P-glycoprotein (P-gp), a transporter responsible for chemotherapy efflux from the cells, highly expressed in PTX-resistant lines.

**Discussion:**

These findings were in line with a greater sensitivity of resistant cells to treatment with both Ramucirumab and Elacridar. Ramucirumab significantly reduced the expression of angiogenic molecules and TUBβIII, while Elacridar restored the access of chemotherapy, recovering its anti-mitotic and pro-apoptotic effects. Finally, this study highlighted the role played by exosomes in spreading factors responsible for resistance in the tumor microenvironment.

## Graphical Abstract

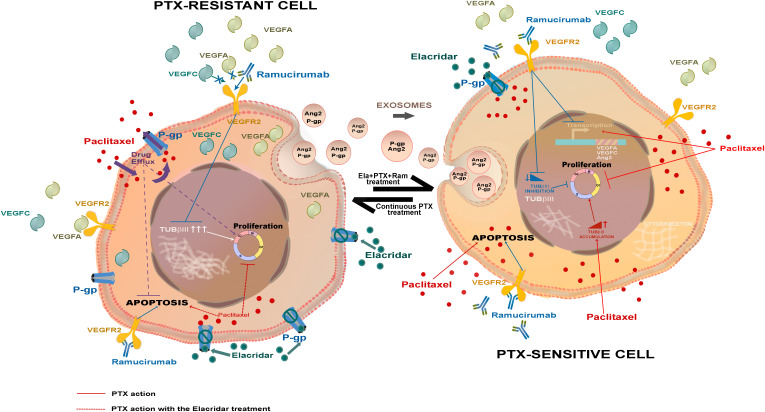

## 1 Introduction

Paclitaxel (PTX) is a taxane belonging to the class of antimitotic drugs with microtubules stabilization action. PTX is known to interfere with microtubules dynamics by binding to β-tubulin, thereby blocking mitotic progression and inducing apoptosis (1). The mechanisms of paclitaxel cytotoxicity are likely to be multifactorial because varying effects are seen in different cell lines, indeed its blocking action could occur at different phases of the cell cycle depending on cell lines (2, 3). PTX exerts its action by promoting tubulin polymerization and inhibiting tubulin depolymerization (1). Microtubules are involved in a variety of cellular processes, such as signaling, migration and division, which are crucial for the growth of cancer cells and metastases. Microtubules disorganization decreased responses to antimitotic treatments and apoptotic process failure can induce acquired resistance to PTX in advanced Gastric Cancer (GC) (4, 5).

In advanced GC, PTX is used in front line chemotherapy schemes including two or more chemotherapy agents and in combination with Ramucirumab in second line therapy (6, 7). Ramucirumab is a monoclonal antibody against the Vascular Endothelial Growth Factor Receptor 2 (VEGFR2) and it is the first anti-angiogenic agent that demonstrates activity for advanced GC. Based on the results obtained by two different phase III studies, Ramucirumab is approved both as monotherapy and in combination with PTX for this malignancy in patients with disease in progression after a preceding therapy based on platinum and fluoropyrimidine (7, 8). The proangiogenic actions of VEGFs in endothelial cells are primarily mediated through binding and activation of VEGFR2 which plays a crucial role in gastric cancer pathogenesis and progression (9, 10). In tumor cells, the VEGFA-VEGFR2 binding activates autocrine survival and migration signaling in an angiogenesis-independent manner. The combined treatment with PTX and Ramucirumab seems to be a promising option to prevent the resistance to PTX also in recurrent and metastatic gastric cancer patients receiving taxane-based first-line palliative chemotherapy (11).

The effectiveness of the treatment that combines PTX and Ramucirumab comes from the cross-talk between the cascades of events on which the two drugs exert their own action. VEGFR2 signaling is involved in several aspects of endothelial cells function, including dynamic changes in endothelial cells shape. The interactions between VEGFR2 and structural proteins may play a pivotal role in VEGFR2 subcellular sorting and signaling (12). It is well known that microtubules inhibitors interfere with VEGF signaling in endothelial cells, pointing to an important role of the cytoskeleton in the signaling and dynamic sorting of VEGFR2. Interestingly, another study demonstrated that blocking VEGFR1/2 receptors may lead to a decrease in Tubulinβ III (TUBβIII) levels (13).

The resistance mechanisms observed in many tumors are due to the overlapping combinations of different determinants of resistance, the analysis of which could provide a useful map for their overall understanding. One of drug resistance mechanisms, known as Multi Drug Resistance (MDR), is mediated by the overexpression of trans-membrane pumps that cause the efflux of therapeutic agents from the cell (14). The P-glycoprotein (P-gp, MDR1), together with its sister proteins Breast Cancer Resistant Protein (BCRP) and Multi Drug Resistance associated Protein 1 (MRP1), is one of the claimed MDR proteins belonging to the super family of the ATP Binding Cassette (ABC) transporters (15). In physiological conditions, P-gp is the gatekeeper of the blood-brain barrier and the gastrointestinal tract, avoiding the access of toxic substances to the brain and gut, but it was found overexpressed in many tumors and its level can significantly in-crease after one or more cycles of chemotherapy (15–17). Several chemotherapy agents including PTX have been identified as P-gp substrates (18). Therefore, the P-gp inhibition may potentially restore PTX activity in PTX-resistant cells. Elacridar represents the mostly known P-gp inhibitor and combined treatments with Elacridar-PTX can rehabilitate the access of chemotherapy to the cell thus restoring its cytotoxicity (19, 20).

Many of the features that confer resistance to drug treatment can be spread in the tumor microenvironment through exosomes. Extracellular communication through exosomes plays a pivotal role in cancer progression, invasion, neovascularization, metastasis and drug resistance (21). These small nano-sized particles can carry or expose on their surface various biomolecules including lipids, proteins, mRNAs, and miRNA and it has been demonstrated that exosomes are secreted in substantially higher concentrations from cancer cells. Exosomes released by cancer cells transfer oncogenic molecules not only to other malignant cells, but also to stromal cells and have the potential to promote transformation of normal cells (22).

The present study aimed to investigate the underlying mechanisms of PTX resistance in human GC cell lines. Crucial aspects such as activation of pro-angiogenic factors, overexpression of MDR transporters, and the spread mechanism of resistance characteristics in the tumor microenvironment have been investigated.

## 2 Materials and methods

### 2.1 Cells and drugs

AGS, HGC27, KATO III and NCI-N87 [N87] human gastric cancer cell lines were purchased and authenticated by American Type Culture Collection (Manassas, Virginia, USA) and HGC27 by Interlab Cell Line Collection (Genova, Italy). All cell culture components were purchased from Sigma-Aldrich (Milan, Italy). PTX was purchased from Teva Italia S.r.l. (Milan, Italy) and Ramucirumab from Eli Lilly (Utrecht, Nederland). Elacridar (GF120918 or GW120918). Elacridar was purchased from Sigma-Aldrich (Milan, Italy).

The concentration of Ramucirumab used in the experiments presented in this study resulted from previous dose-response experiments performed with Ramucirumab and PTX administrated alone and in combination. These data revealed that 100 µg/mL Ram enhanced the inhibition exerted by concentrations of PTX close to its IC50. According to Chou e Talalay method, the Combination Indexes (CI) obtained for these drug combinations were ≤1, demonstrating that drugs exerted their effect synergistically in both HGC27 and KATOIII cell lines (23).

### 2.2 Cell culture

Gastric cancer cell lines were cultured in Dulbecco’s Modified Eagle Medium (DMEM) supplemented with 10% of Inactivated Exosome depleted Fetal Bovine Serum (FBS) exosomes free (Euroclone), 100 U/mL penicillin and 100 µg/mL streptomycin. The cells were incubated at 37 °C in a humidified atmosphere containing 5% CO2 atmosphere.

### 2.3 Generating Paclitaxel-resistant HGC27 and KATOIII cell lines

Paclitaxel-resistant HGC27 and KATOIII lines (HGC27-R and KATOIII-R) were obtained by exposure to increasing concentrations of the taxane, starting with a concentration of 1/60th of the IC50 and continuing with subcultures by increasing the concentration by 25% every fortnight (24). Cells are defined as resistant when they are able to grow exponentially in the presence of a Paclitaxel concentration equal to that of the IC50 of the sensitive counterpart. HGC27-R and KATOIII-R were grown under the same experimental conditions as their sensitive counterpart with the addition of PTX at 9 nM and 3 nM respectively. All experiments comparing resistant and sensitive cell lines were performed at a PTX concentration close to the IC50 of the sensitive cells. **Figure S1** shows the values of the half-maximum inhibitory concentration (IC50) obtained by exposing the cells to increasing dosages of PTX for 48 hours. It also highlighted the altered cell morphology induced by resistance to PTX. Microscopy images showed that while HGC27-S cells appeared elongated with a clear boundary the HGC27-R cells that were instead polygonal and grouped (**Figure S1**). KATOIII cells grow partly in adhesion and partly in suspension. Therefore, differences between sensitive and PTX resistant cells showed less appreciable differences. Nevertheless, also in this case the contours of the KATOIII-S cells appeared more defined and regular than those of KATOIII-R. The latter also grew in a greater proportion in suspension.

### 2.4 Gene expression analysis

Total RNA was extracted from HGC27 and KATO III cells using the Qiagen RNeasy Mini Kit (Qiagen, Hilden, Germany) according to the manufacturer’s instructions. Samples were retro-transcribed using the iScript Advanced cDNA Synthesis Kit (Bio-Rad Laboratories, California, USA). cDNA samples from HGC27-S/R and KATOIII-S/R were analyzed using PrimePCR pre-constituted assays from Bio-Rad to study genes differentially expressed within the angiogenesis pathway and specifically within VEGF/VEGFR signaling. Results for some of the genes differentially expressed in both lines were validated using specific Primers. The SsoAdvanced PreAmp Supermix and PrimePCR PreAmp for SYBR Green Assay (Bio-Rad Laboratories, California, USA) were used as indicated in the users’ guide to provide unbiased preamplification reactions regardless of KDR, enabling more copies of genes to be obtained from a limited source. Real Time-PCRs for the evaluation of VEGFA, VEGFC, KDR and ANGPT2 expression were carried out in triplicate using the SsoAdvanced Universal SYBR Green Supermix (Bio-Rad Laboratories, California, USA) on a CFX96 Touch Real-Time PCR Detection System (Bio-Rad Laboratories, California, USA) according to the manufacturer’s instructions. The mRNA expression was normalized on GAPDH housekeeping gene. Pre‐validated PrimePCR Template for SYBR Green Assay (Bio-Rad Laboratories, California, USA) were used for reactions. Relative quantification was done using the ddCT method.

### 2.5 STRING program analysis

The differentially expressed genes in the PTX-resistant cell lines with respect to the sensitive ones were included in the STRING program (STRING: functional protein association networks (string-db.org)) known as predictor of protein-protein interactions (**Figure S2**). The interactions included direct (physical) and indirect (functional) associations. Interactions in STRING are derived from five main sources: Genomic Context Predictions, High-throughput Lab Experiments, (Conserved) Co-expression, Automated Textmining, Previous Knowledge in Databases.

### 2.6 Western Blotting analysis

Western Blotting analysis was performed as previously described (25) in both HGC27-S/R and KATOIII-S/R. Briefly, for each experimental condition the cells were lysed in ice-cold lysis buffer (50 mM Tris, 10 mM EDTA, 1% v/v Triton-X100), supplemented with the protease/phosphatase inhibitor cocktail set (Merck KGaA, Darmstadt, Germany), incubated on ice for 15 minutes and centrifuged at 13,000 × g for 15 min at 4°C. Separate cytoplasmic and nuclear protein fractions were obtained using the NE-PER Nuclear and Cytoplasmic Extraction Kit (Thermo Fisher Scientific Inc., MA USA) following the manufacturing procedures. Protein extracts were quantified by Micro BCA™ Protein Assay Kit (Thermo Fisher Scientific Inc., MA USA) and 40 μg of total protein extract or nuclear/cytoplasmic fraction were subjected to SDS-PAGE and immunoblotted with the following antibodies: P-gp (1:250, rabbit polyclonal, Santa Cruz Biotechnology Inc., Santa Cruz, CA), MRP1 (1:500, mouse clone MRPm5, Abcam, Cambridge, UK), BCRP (1:500, mouse clone BXP-21, Santa Cruz Biotechnology Inc.), Phospho-βcatenin (Ser675) and βcatenin (1:1000 Cell Signaling, Beverly, MA, USA), NFkB (1:1000, Santa Cruz Biotechnology Inc.), Phospho-cyclin B1 (Ser147) and cyclin B1 (1:1000 Cell Signaling, Beverly, MA, USA), Beta III Tubulin (TUBβIII 1:2000 Abcam, Cambridge, UK), Phospho-c-Myc (Ser62) and c-Myc (1:1000 Cell Signaling, Beverly, MA, USA), Phospho-SAPK/JNK (Thr183/Tyr185) and JNK2 (1:1000 Cell Signaling, Beverly, MA, USA), Phospho-c-Jun (Ser63) and c-Jun (1:1000 Cell Signaling, Beverly, MA, USA), caspase 3/7 (1:1000 Cell Signaling, Beverly, MA, USA), VEGF Receptor 2 (VEGFR2 1:500, Santa Cruz Biotechnology Inc., Santa Cruz, CA) VEGF receptor 3 (VEGFR3 1:200, Abcam, Cambridge, UK), Angiopoietin 2 (Ang 2 1:500, R&D Systems, Minneapolis, MN, USA), VEGFA (Abcam, Cambridge, UK), Fibronectin1 (FN1 1:400; Invitrogen), ALIX (1:1000; Abcam, Cambridge, UK), CD81 (1:500; Invitrogen) and GAPDH (1:1000 Abcam, Cambridge, UK). Subsequently, the membranes were incubated with the corresponding horseradish peroxidase (HRP)-conjugated secondary antibodies (Bio-Rad, Hercules, CA, USA). An enhanced chemiluminescence kit (Bio-Rad, Hercules, CA, USA) was used. A Chemidoc XRS+ and the Bio-rad software (Bio-Rad, Hercules, CA, USA) was used to observe and analyze the chemiluminescence signals from proteins. Nuclear protein extracts have been normalized using stain free technology, using Image Lab Software (Bio-Rad, Hercules, CA, USA). Total protein expression was quantified using the ImageJ software (http://rsb.info.nih.gov/ij/).

### 2.7 [^3^H] Paclitaxel accumulation

1×10^5^ cells were incubated 1, 3, 6 and 24 hrs with 10 or 100 nM Elacridar plus 1µCi [^3^H] PTX (co-incubation setting) or pre-incubated for 24 hrs with 10 or 100 nM Elacridar, followed by 1µCi [^3^H] PTX for 1, 3, 6 or 24 hrs (pre-incubation setting). After this incubation time, cells were washed five times with PBS, detached by gentle scarping and resuspend in 2 mL of scintillation liquid (PerkinElmer). A 50 µL aliquot of cell suspension was sonicated and used to determine the intracellular protein content. The radioactive content of [^3^H] PTX, inversely related to the efflux of PTX, was counted by a β-counter (PerkinElmer). For each experimental condition, a sample without [^3^H] PTX was counted and used as a control of background radioactivity. The count per minutes (cpm) was converted into nM [^3^H] PTX/mL, according to a titration curve previously prepared. The results were expressed as nM [^3^H] PTX/mg cellular proteins.

### 2.8 Cytotoxicity assay

The assay was performed in HGC27-S/R and KATOIII-S/R cells 48 hrs upon treatment, as already reported with a minor modification (26). Briefly, the cells were seeded into 96-well plates at density of 15000 cells/well in a volume of 100 μL of fresh medium. Next day Elacridar (10 nM, 100 nM, 1 μM) and PTX (4 nM for HGC27 and 2 nM for KATOIII) were added to the cells alone and in combination. The plates were incubated for 48 hrs, in a humidified incubator at 37 °C with a 5 % CO2 atmosphere. After the incubation time, 3-(4,5-dimethylthiazol-2-yl)-2,5-diphenyltetrazolium bromide (MTT) was added to each well, and after 3-4 hrs incubation at 37 °C, the supernatant was removed. The formazan crystals were solubilized using 100 μL of DMSO/EtOH (1:1), and the absorbance values at 570 and 630 nm were determined on the microplate reader Victor 3 from PerkinElmer Life Sciences (Mechelen, Belgium). Dose-response curves were calculated for each drug or drug combination and relative IC_50_ values were computed using Microsoft Office Excel. Each value was calculated from equation y = mx + c derived from the dose-response curve (with y = 50, the equation became: 50 = slope*IC50 + intercept).

### 2.9 Cell cycle assays

#### 2.9.1 Muse Cell Cycle assay

The cell cycle was analyzed using the Muse Cell Cycle kit (Millipore, Darmstadt, Germany) with and without G2/M phase synchronization obtained by treatment with nocodazole. HGC-27-S/R and KATOIII-S/R cells were synchronized by using 20 µg/mL nocodazole added to the medium. After 18 hrs of incubation, the medium containing nocodazole was replaced from fresh medium and cells were separated into two groups: one group was collected for cell cycle analysis (T0) and the second one continued culturing for 9 more hours as previous described (T1) (**Figure S3**). The same pharmacological treatments were administered also to non-synchronized cells for 12 hrs. After incubation with drugs, the cells were processed by Muse Cell Cycle Kit according to Muse Cell Analyzer protocol that determined the population of cells in the different phases of cell cycle. The percentage of cells in each cell cycle phase was reported in the relative graphs.

#### 2.9.2 Cell-Clock™ Cell Cycle assay

In alternative, cell cycle was assessed also using the Cell-Clock™ Cell Cycle Assay (Biocolor Life Science Assay, Carrickfergus County Antrim, UK), a live cell detection and measurement system useful to monitor the cell cycle phases during *in vitro* cultures. This assay uses a redox dye uptake by the cycling cells, after the dye uptake and incubation, a distinct color change occurs within cells and these changes are associated to cells in the G0/G1 (yellow staining), S (green staining), G2/M (dark green/blue staining) phases. Briefly, the cells were seeded into the 24-well plate at a density of 2 x 10^4^ cells/well in DMEM or RPMI culture medium (Gibco); after 24 hours, the cells were treated with or without Elacridar in combination or not with PTX (4 nM for HGC-27-S/R and 2 nM for KATOIII-S/R cells). After 12 hrs, the medium was replaced with 500 μL of fresh medium. Furthermore, 150 μL of cell-clock dye reagent was added on each well and incubated for 1 hour at 37 °C. The dye reagent was removed from each well and, after washing with fresh medium, replaced with 200 μL fresh medium. Images were acquired of live cells and analyzed by means of an Eclipse Ti2 Nikon confocal microscope. All images were taken at 20× magnification. The calculation of phase ratios was obtained by computer software analysis (Image J) of the digitized images of photomicrographs (**Figure S4**).

### 2.10 Apoptosis assay

HGC27-S/R and KATOIII-S/R were treated with PTX (4 nM for HGC-27-S/R and 2 nM for KATOIII-S/R cells), 100 μg/mL Ramucirumab and 100 nM Elacridar administrated alone or in combination for 48 hrs. After specified drug treatments the cells were processed by the Muse Annexin V/Dead Cell Assay Kit (Millipore, Darmstadt, Germany) for quantitative analysis of live, early/late apoptotic and dead cells was used with a Muse Cell Analyzer. Briefly, the assay utilizes Annexin V to detect phosphatidyl serine on the external membrane of apoptotic cells. The fluorescent signal emitted by dye conjugated antibodies was detected by Flow cytometry technology (Muse Cell Analyzer, Millipore, Darmstadt, Germany). 7-Amino-Actinomycin D (7-AAD) dead cell marker is also used. The cells were then analyzed as described in the user’s guide.

### 2.11 Migration assay

HGC27-S/R grew for 48 hrs in medium containing PTX, Ramucirumab, Elacridar alone or in combination and solvent (control), until confluence. A scratch wound was generated with a pipette tip. After rinsing with medium to remove detached cells, low serum medium (1% FBS) without drug was added. Photographs were taken of each well immediately (T0) and after various times T1 (24 hrs), T2 (48 hrs), and T3 (72 hrs), using a Leica DMRXA camera (Leica Microsystems, Milan, Italy). Images were analyzed using ImageJ Software (http://rsb.info.nih.gov/ij/). The distance that cells migrated through the area created by scratching was determined by measuring the wound width at T1, T2 and T3 and subtracting it from the wound width at the start. The relative migration rate was calculated by setting the percentage of migration of the control cells at time T1 equal to 1 and comparing the percentage of migration of the cells after each drug treatment to this value. The results were representative of three independent experiments.

### 2.12 Measurement of VEGFA and VEGFC in cell culture medium

The amount of VEGFA and VEGFC secreted in the culture medium by HGC27-S/R and KATOIII-S/R cells was measured using highly sensitive Enzyme-Linked Immunosorbent Assay (ELISA) Quantikine Kit ELISA (R&D Systems, Minneapolis, MN, USA) according to the manufacturer’s instructions. The measured values were normalized for the number of cells.

### 2.13 Exosomes isolation

Medium derived from both HGC27-R and KATOIII-R was processed for exosome isolation, in according to a previously published procedure (27) and as reported in the MISEV 2018 guidelines (28). Briefly, the culture medium of cells was centrifuged for 10 minutes at 1500× g (Thermo Scientific, Heraeus Multifuge X3 Centrifuge). The supernatant was transferred to a clean tube and centrifuged again at 1800× g for 10 min. Subsequently, the supernatant was carefully transferred into a sterile tube and further centrifuged first at 3000× g for 15 minutes and then at 3800× g for 15 minutes. The super-natant was ultra‐centrifuged at 75,000× g for 2 hours (BECKMAN, L‐60 Ultracentrifuge) and, the supernatant again ultra‐centrifuged at 100,000× g for 2.5 hrs. All centrifugation steps were carried out at 4 °C. The pellet consisting of purified exosomes was recovered and dispersed in 200 μL of ultrapure water for the chemical-physical characterization and protein extraction or in sterile PBS for the cultured cell. After the exosome extraction, a characterization of freshly isolated exosome samples was performed by Dynamic Light Scattering (DLS) analysis, ς-Potential measurements and Transmission Electron Microscopy (TEM) investigation as previously reported (27). The remaining sample was stored at -80°C until protein extraction was assessed.

### 2.14 Protein quantification and analysis of medium-derived exosomes

Western blotting analysis was performed on the total protein content extracted from HGC27-S and KATOIII-S cells previously treated with exosomes derived from the conditioned medium of the cells of their respective PTX-resistant counterpart. The same analysis was performed to compare the content of specific proteins in exosomes derived from PTX-sensitive (EXO-S) cells to that derived from PTX-resistant cells (EXO-R). Untreated cells were used as control. Briefly, 1 × 10^6^ cells were used for each experimental condition of treatment with or without exosomes. The cells were treated daily with exosomes derived from resistant cell lines and after 96 hrs the total proteins were extracted as described above.

### 2.15 Cell viability assay

Both HGC27-S and KATOIII-S were treated with exosomes derived from HGC27-R and KATOIII-R respectively (referred as EXO-R) and PTX at two different concentrations (4 nM and 8 nM for HGC27 and 4 nM and 6 nM for KATOIII). EXO-R and PTX were administrated alone or in combination. Briefly, cells were seeded into 96-well plates at a density of 2 × 10^3^ cells/well. After 24 hrs, the cells were treated with an amount of EXO-R that in terms of total protein content was equivalent to 20 µg and/or with PTX for 48, 72 and 96 hrs. After the incubation time, the cells were treated with the MTS tetrazolium compound (CellTiter 96® AQueous One Solution Cell Proliferation Assay, Promega) for three additional hours and the absorbance was measured at a wavelength of 490 nm using a Perkin Elmer Victor Plate Reader (Mechelen, Belgium).

### 2.16 Statistical analysis

GraphPad Prism 5.0 software (La Jolla, CA, USA) was used to evaluate the differences between two unmatched groups by Mann–Whitney nonparametric test. P<0.05 was considered statistically significant. All experiments were performed in triplicate and repeated three times. Data were presented as mean ± standard deviation (SD).

## 3 Results

### 3.1 Overexpression of VEGF/VEGFR family molecules as a hallmark of PTX resistance

Differentially expressed genes in PTX-resistant cell lines versus sensitive ones have been identified within a pre-constituted panel of 88 genes involved in angiogenesis. The 31 deregulated genes in PTX-resistant cells (HGC27-R and KATOIII-R) compared to PTX-sensitive ones (HGC27-S and KATOIII-S) are shown in **Table 1**. Genes with a fold increase ≥ 2 were considered up-regulated and those with a fold increase ≤ -2 were considered down-regulated. Over-expressed genes included growth factors such as the VEGF family genes VEGFA, VEGFC, FLT4 (VEGFR3) or closely related genes such as Angiopoietin 1 and 2 (ANGPT1 and ANGPT2). Down-regulated genes included those encoding growth factor receptors such as KDR (VEGFR2), Platelet-Derived Growth Factor Receptor Beta (PDGFRB) and Transforming Growth Factor Beta Receptor 2 (TGFBR2), as well as the gene for the Connective Tissue Growth Factor (CTGF). **Figure S2** shows the relationships between the studied angiogenic markers obtained with the STRING program. In this complex network, members of the VEGF/VEGFR family play a key role and have been further investigated. Other genes differentially expressed in PTX-resistant lines were not further examined in the present study.

**Table 1:** Thirty-one deregulated genes in PTX-resistant cells (HGC27-R and KATOIII-R) compared to PTX-sensitive ones (HGC27-S and KATOIII-S) were shown.

Target	HGC27-R *vs.* -S Fold Change	P Value	KATOIII-R *vs.* -S Fold Change	P Value	Target Regulation
**AKT3**	4,42	0,004	4,64	0,0012	Up regulated
**ANGPT1**	6,38	0,002	4,67	0,002	Up regulated
**ANGPT2**	4,16	0,0013	2,64	0,006	Up regulated
**ANPEP**	-8,92	0,004	-2,74	0,015	Down regulated
**ANXA2**	-4,20	0,005	-2,81	0,02	Down regulated
**BMP4**	-9,83	0,003	-24,89	0,001	Down regulated
**CAV1**	-3,00	0,023	-3,39	0,0034	Down regulated
**CCL2**	-4,13	0,0015	-4,91	0,0022	Down regulated
**CD34**	145,19	0,0008	9,15	0,0019	Up regulated
**CTGF**	-2,47	0,022	-3,67	0,005	Down regulated
**CXCR2**	8,89	0,003	216,10	0,0012	Up regulated
**CYR61**	-2,69	0,019	-4,17	0,004	Down regulated
**EDNRA**	-4,10	0,005	-3,75	0,007	Down regulated
**EPHA2**	-2,74	0,032	-2,45	0,009	Down regulated
**EPHB2**	-2,33	0,021	-2,57	0,0063	Down regulated
**FLT4**	5,00	0,007	2,10	0,021	Up regulated
**FN1**	17,56	0,001	10,47	0,007	Up regulated
**IL18**	-9,47	0,0014	-9,14	0,004	Down regulated
**ITGA2**	12,97	0,003	5,26	0,016	Up regulated
**KDR**	-3,72	0,02	-2,10	0,18	Down regulated
**MMP14**	-2,49	0,034	-2,30	0,032	Down regulated
**NRP1**	-3,87	0,052	-2,63	0,03	Down regulated
**PDGFRB**	-5,88	0,002	-4,31	0,002	Down regulated
**PIK3CG**	2,72	0,031	3,21	0,0042	Up regulated
**PTEN**	4,75	0,0016	2,89	0,018	Up regulated
**PTGS2**	38,34	0,0012	38,98	0,0013	Up regulated
**SERPINE1**	-2,74	0,025	-3,10	0,0033	Down regulated
**TGFBR2**	-3,95	0,0063	-3,33	0,006	Down regulated
**THBS1**	-10,53	0,002	-22,04	0,004	Down regulated
**VEGFA**	2,44	0,004	2,39	0,62	Up regulated
**VEGFC**	5,42	0,01	2,52	0,02	Up regulated

Genes with a fold increase ≥ 2 were considered up-regulated and those with a fold increase ≤ -2 were considered down-regulated. Unpaired t test was performed, P value ≤ 0,05 was considered statistically significant.

**Figure 1:**
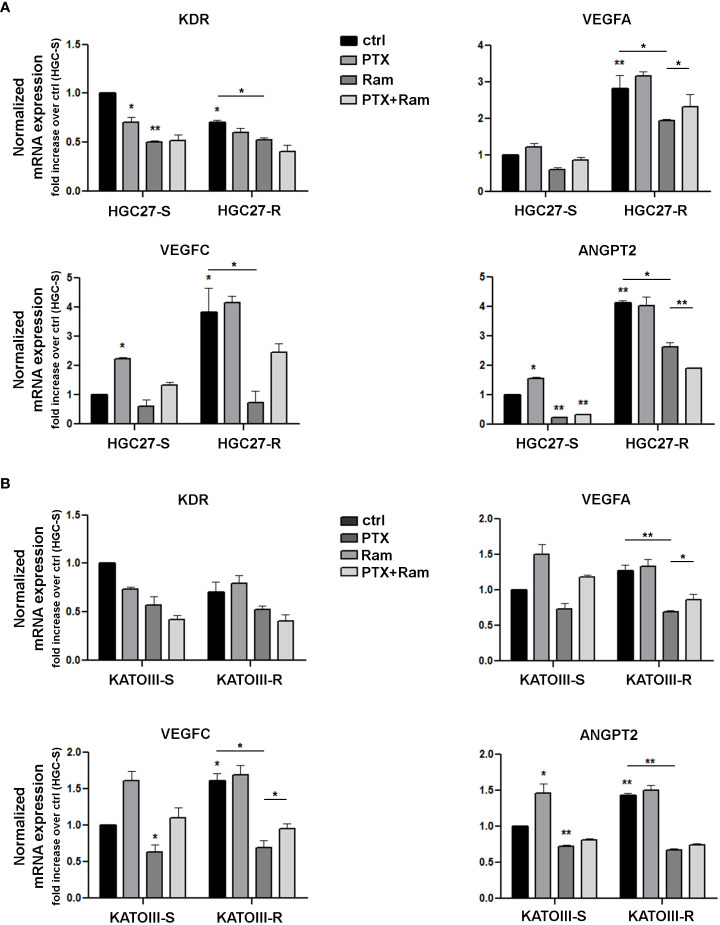
Real time PCR experiments in HGC27-S/R (A) and KATOIII-S/R (B) with specific primers for KDR, VEGFA, VEGFC, ANGPT2 genes. The mRNA expression was normalized on GAPDH housekeeping gene. Expression analysis performed after treatment with 4 nM PTX (HGC27) or 2 nM PTX (KATOIII) and 100 mg/mL Ramucirumab administered individually or in combination for 24 hrs. All expression values were calculated against the value of untreated PTXsensitive cells, fixed at 1. Statistical analysis was assessed comparing the values obtained using single drug treatment to those of corresponding untreated cells and the combined treatments to those of single treatments. The values of untreated PTX-resistant cells were compared with those of untreated PTX-sensitive ones. Data were mean ± SD (n=3). *p<0.05, **p<0.01.

The results obtained for members of the VEGF/VEGFR family were validated with real time experiments using KDR, VEGFA, VEGFC, ANGPT2 specific primers. Expression analysis was conducted in both lines of HGC27-S/R (**Figure 1**, panel A) and KATOIII-S/R (**Figure 1**, panel B) after treatment with 4 nM (HGC27) or 2 nM (KATOIII) PTX and 100 μg/mL Ramucirumab administered individually or in combination. PTX treatment resulted in a decrease in KDR and an increase in VEGFA, VEGFC and ANGPT2 in sensitive cells, while Ramucirumab treatment, in the same cells, determined a decrease in the expression levels of all the genes investigated. The combined treatment did not lead to a further significant decrease in KDR. Furthermore, the contrasting actions of the two aforementioned drugs, caused an increase in the expression of the VEGFA, VEGFC and ANGPT2 markers when compared to a single Ramucirumab treatment. However, the combined treatment with those two drugs had a decrease of said markers compared to the control cells. Although in sensitive cells the increase with PTX and the decrease with Ramucirumab are not statistically significant, in cells in which resistance to PTX was induced, the levels of VEGFA and VEGFC and ANGPT2 are significantly higher than in the sensitive counterpart. Therefore, in both resistant lines, the high levels of growth factors may be related to an increased activation of this signaling pathway, resulting in a much stronger inhibitory action of Ramucirumab. In these cells, the decrease in the expression levels of the three growth factors compared to sensitive cells was always significant, while PTX treatment was ineffective in modifying their expression levels. The combined treatment resulted in a further reduction in the expression levels of KDR and ANGPT2 but not of those of VEGFA and VEGFC.

### 3.2 Elacridar restores the inhibitory action of Paclitaxel on cell growth in resistant GC cells expressing high levels of P-gp

**Figure 2:**
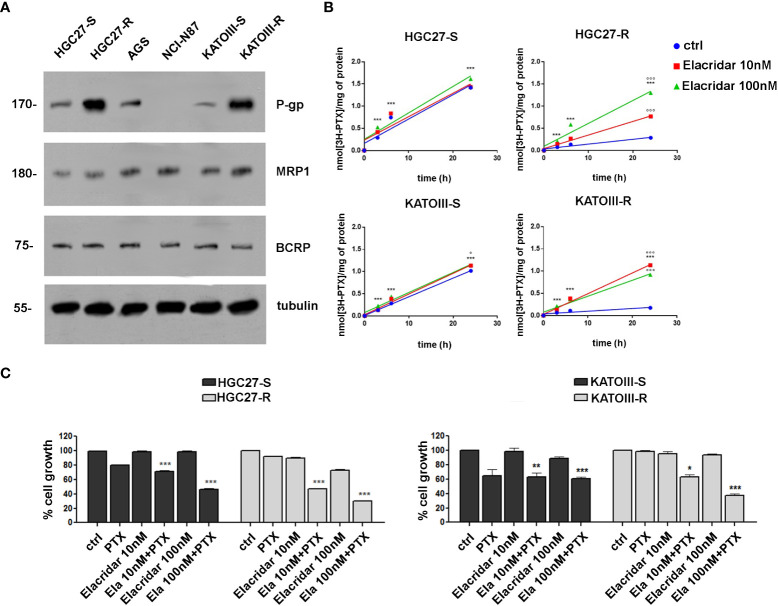
**(A)** Immunoblotting of ABC transporters in GC cell lines (HGC27-S/R, AGS, NCl-N87 and KATOIII-S/R). Whole cell lysates were subjected to immunoblotting for the indicated proteins. Tubulin was used as control of equal control loading. The figure is representative of 3 independent experiments; **(B)** accumulation of [3H]-PTX. HGC27-S/R and KATO-S/R were co-incubated for 1, 3, 6 and 24 hrs with 10 or 100 nM Elacridar plus 1 μCi [3H]-PTX (co-incubation setting). The intracellular amount of [3H]-PTX was counted by liquid radio scintillation. Data are mean ± SD (n=3). Statistical analysis was assessed comparing each experimental condition to its own time 0, considered as a blank, (***p<0.001); The values obtained following treatment with Elacridar 10 nM and 100 nM for 24 hrs were compared to the value obtained at the same time in untreated cells, °°°p<0.001, °p<0.05; **(C)** Cytotoxic effect at 48 hrs of PTX (2 nM) and Elacridar (10 nM and 100 nM) alone and in combination in HGC27-S/R and KATOIII-S/R cell lines. Statistical analysis was assessed comparing the values obtained using single drug treatment to those of corresponding untreated cells, considered as 100, and the combined treatments to those of the single treatments. Data were mean ± SD (n=3). *p<0.05, **p<0.01, ***p<0.001.

The expression levels of MDR proteins such as P-gp, MRP1 and BCRP were investigated in PTX-sensitive GC lines (HGC27-S, KATOIII-S, AGS, NCl-N87) and in two lines of HGC27 and KATOIII resistant to PTX action (HGC27-R and KATOIII-R), obtained from their sensitive counterparts, as described in the methods section. As shown in panel A of **Figure 2**, the P-gp protein is expressed in all cell lines examined with the exception of NCl-N87 and was also significantly over-expressed in both PTX-resistant cell lines (HGC27-R and KATOIII-R). By contrast, no significant differences were found in the expression levels of MRP1 and BCRP. Therefore, the PTX-sensitive and PTX-resistant lines HGC27 and KATOIII were used as an experimental model to investigate the role of P-gp in the efflux of PTX from the cell and consequently in the acquired drug resistance. The correlation between the different expression of P-gp and different intracellular retention of PTX was explored. Both HGC27-S/R and KATOIII S/R cells were radio-labelled with [^3^H]-PTX. After cell washing the intracellular PTX amount was determined in the absence or in the presence of the P-gp inhibitor Elacridar, to mimic an active and blocked P-gp, respectively. In a first experimental set, a fixed concentration of [^3^H]-PTX was co-incubated with 10 nM and 100 nM Elacridar for 1, 3 and 6 hrs. In a second experimental set, Elacridar was pre-incubated 24 hrs before adding [^3^H]-PTX. In both experimental sets there was a time-dependent intracellular accumulation of [^3^H]-PTX, index of reduced efflux. The maximal efficacy was obtained after 6 hrs and with 100 nM Elacridar. Moreover, the co-incubation setting (**Figure 2**, panel B) displayed a greater intracellular accumulation of [^3^H]-PTX than the pre-incubation setting (not shown). This difference can be explained by the competitive nature of Elacridar on P-gp that makes it more effective when it is present with the drug substrate of P-gp. The pre-incubation eliminates part of Elacridar-P-gp complex and allow the cells to resynthesize new P-gp, increasing the efflux of PTX. We detected an increase in the intracellular accumulation of [^3^H]-PTX also in the HGC27 and KATOIII sensitive cell lines, due to their constitutively low levels of P-gp that can be inhibited by Elacridar. Given the low amount of P-gp in sensitive cells, Elacridar did not find its target. Therefore, it exerts no or little additional benefit in HGC27-S and KATOIII-S cells. By contrast, the maximal benefits of Elacridar were observed in HGC27-R and KATOIII-R, showing the highest levels of P-gp, as expected, producing the maximal effects in terms of intracellular PTX retention and toxicity (**Figure 2**, panel b). The efficacy of Elacridar in restoring the inhibitory effects of PTX on cell growth was analyzed by comparing the growth of PTX-sensitive HGC27 and KATOIII cells to that of their resistant counterparts in the presence of PTX (4 nM for HGC27and 2 nM for KATOIII) and Elacridar (10 nM and 100 nM) administered both alone and in combination. As showed in panel C of **Figure 2**, the cytotoxic effects of Elacridar in HGC27-S and KATOIII-S cells were negligible at both concentrations (10 and 100 nM), whereas in the presence of PTX, the cytotoxic effect was recovered at both concentrations, about 30% and 50% at 10 nM and 100 nM Elacridar, respectively in HGC27-S and about 40% and 50% at 10 nM and 100 nM Elacridar, respectively in KATOIII-S. By contrast, in resistant cells, with high expression of P-gp, Elacridar had low cytotoxicity per se (10%), which in the presence of 2 nM PTX was significantly enhanced, near 50% at 10 nM and 70% at 100 nM Elacridar in HGC27-R and 40% at 10 nM and 70% at 100 nM Elacridar in KATOIII-R.

### 3.3 Ramucirumab and Elacridar restore the inhibitory action of Paclitaxel on cell cycle progression in resistant GC cells

**Figure 3:**
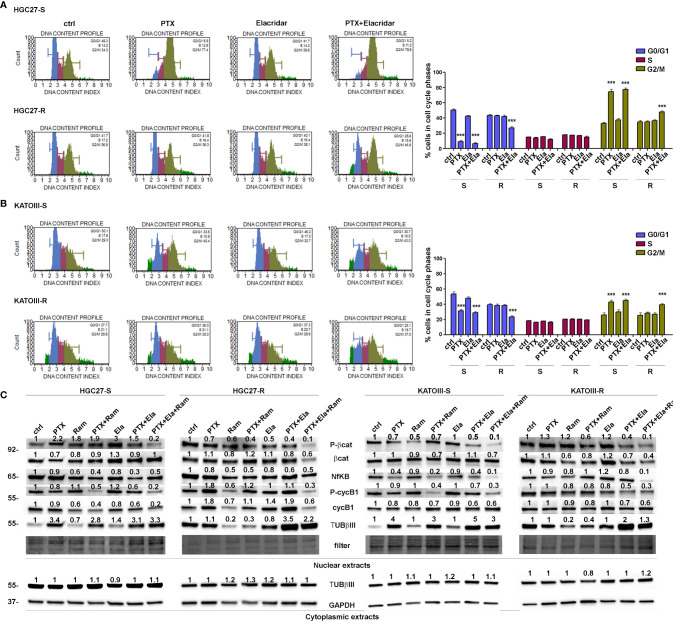
Effects of PTX, Ram and Ela on cell cycle progression in GC cell lines. HGC27-S, HGC27-R (A) and KATOIII-S, KATOIII-R (B) human GC cells were treated with/without 4 nM (HGC27) or 2 nM PTX (KATOIII) and 100 nM Ela alone or in combination for 12 hrs. The percentage of cells in each cell cycle phase characterized by different DNA content index (blue for G0/G1 phase, magenta for S phase and green for G2/M phase) were shown in representative DNA content profiles. Apoptotic cells in sub-G1 (on the left end) or small aggregates of cells (on the right end) were indicated in bright green. The percentage of cells in G0/G1, S and G2/M phases was evaluated for both HGC27 S/R and KATOIII S/R, and plotted in the relative graphs. The results were relative to three independent experiments, expressed as mean ± SD. In each cell cycle phase, statistical analysis was assessed comparing the values obtained in each experimental condition to those of corresponding untreated cells, ***p<0.001. (C) Representative Western Blotting analysis performed in HGC27-S/R and KATOIII-S/R cells relative to the nuclear expression of activated b -catenin, NFkB, Cyclin B1 and TUBbIII and cytoplasmic expression of TUBbIII after 48 hrs of PTX, Ram and Ela single or combined treatments. Nuclear protein extracts have been normalized using stain free technology. GAPDH has been used as a normalizer of cytoplasmic protein extracts. Values indicating normalized expression levels are shown on each band.

**Figure 4:**
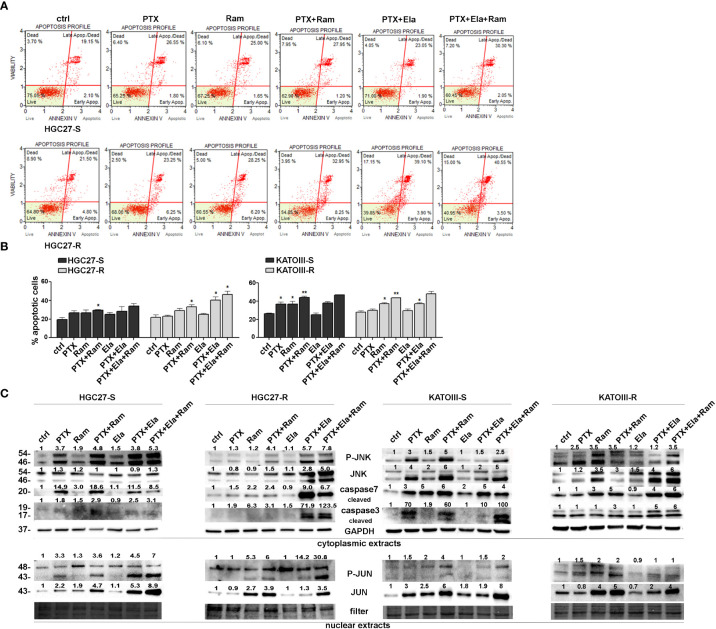
Apoptotic effects of PTX, Ram and Ela in GC cell lines. **(A)** Muse Annexin V Cell Assay for HGC2 S/R and KATOIII S/R cell lines assessed after 48 hrs of 2 nM (for KATOIII) or 4 nM (for HGC27) PTX, 100 μg/mL Ram and 100 nM Ela alone or in combination treatments. In the panel, the results obtained for HGC27-S/R were shown; **(B)** Apoptosis results HGC27 S/R and KATOIII S/R deriving from three independent experiments were expressed as means ± SD and reported in the relative graphs. Statistical analysis was assessed comparing the values obtained using single drug treatment to those of corresponding untreated cells and the combined treatments to those of the single treatments, *p < 0.05; **p < 0.01; **(C)** Representative Western Blotting analysis performed in HGC27 S/R and KATOIII cells relative to the nuclear expression of P-JUN/JUN and the cytoplasmic expression of P-JNK/JNK and cleaved Caspase 3/7. Nuclear protein extracts have been normalized using stain free technology. GAPDH has been used as a normalizer of cytoplasmic protein extracts. Values indicating normalized expression levels were shown on each band.

Cell distribution analysis at different phases of the cell cycle was performed by cytofluorometer method on HGC27-S/R and KATOIII-S/R after treatment with Paclitaxel, affecting the cell cycle directly, and Elacridar, which increased the concentration of intracellular PTX mainly in PTX-resistant cells. Representative DNA content profiles are shown in panels A (HGC27) and B (KATOIII) of **Figure 3**; the percentages of HGC27 and KATOIII cells blocked at different phases of cell cycle after each drug treatment are reported in the corresponding graphs. In both HGC27-S and KATOIII-S cells, PTX treatment (4 nM for HGC27 and 2 nM for KATOIII), compared to untreated cells, resulted in a decrease in the percentage of cells in G0/G1 (8.9% *Vs.* 50.4% in HGC27-S and 31.2% *Vs.* 53.4% in KATOIII-S) which was matched by an increase in that in G2/M (74.5% *Vs.* 32.6% in HGC27-S and 42.9% *Vs.* 25.8% in KATOIII-S). No significant variations were evident in the percentage of cells in S-phase. Treatment with 100 nM Elacridar showed no effect on the percentage of cells at different stages of the cycle compared to untreated cells. The combined treatment, as expected in sensitive cells, showed just a slight increase in the rate of cells in S-phase, compared to the one with PTX alone. By contrast, in both resistant lines and in the same experimental conditions, while the single treatments with PTX and Elacridar produced no change on cell cycle progression compared to untreated cells, the combined PTX-Elacridar treatment partially restored the blocking of cells in G2/M (47.6% *vs.* 34.6% in HGC27-R and 39.2% *vs.* 28.2% in KATOIII-R) with the corresponding decrease of cells in G0/G1 (26.6% *vs.* 42.9% in HGC27-R and 23.1% *vs.* 38.1% in KATOIII-R) (**Figures 3A, B**). To additionally support the data shown in **Figure 3**, prior the cell cycle analysis, the cells were synchronized in G2/M by treatment with 20 nM nocodazole (T0) and then subjected to the drug treatments for 9 hrs, upon the removal of nocodazole, the treatment with PTX resulted in a higher fraction of cells blocked in G2/M (26.6% *Vs.* 42.9% in HGC27-R and 30% *Vs.* 20% in KATOIII-R) compared to untreated cells (T1) in both sensitive cell lines. Also in these experimental conditions, the effects of PTX on the cell cycle were significant only after combined treatment with Elacridar and PTX in both resistant lines (41.7% *Vs.* 28.6% in HGC27-R and 38.8% *Vs.* 30.5% in KATOIII-R) as shown in **Figure 3S**, panel A and B. All these results were confirmed assessing cell cycle progression with the Cell-Clock™ Cell Cycle Assay, a system for detecting and measuring live cells in the different phases of the cell cycle after each drug treatment described above. For each cell line investigated, representative images of live cells stained in the colors corresponding to the different phases of the cell cycle and graphs of the percentage of cells in each phase are shown in **Figure 4S**, panels A and B.

Furthermore, the effects of both Elacridar and Ramucirumab in restoring the inhibitory effect of PTX on the expression of transcription factors actively involved in cell cycle progression and thus in the proliferative process were investigated. The combined treatment of Ramucirumab and PTX resulted in a significant reduction in the nuclear expression levels of active forms of β-catenin, NFkB and CyclinB1 (cycB1) in both sensitive end resistant cells. In PTX-resistant cells there was a further decrease in the expression of these transcription factors with the simultaneous addition of Ramucirumab, PTX and Elacridar. In addition, the analysis of TUBβIII expression in the nuclear fraction showed that treatment with PTX resulted in a significant increase in this protein expression in drug-sensitive cell lines. Combined treatment with Ramucirumab or Elacridar did not induce further changes in its expression levels. Also, both single treatments with Ramucirumab or Elacridar did not cause significant changes of TUBβIII expression in the nuclear fraction of PTX-sensitive cells compared to untreated cells. In PTX-resistant cells, an accumulation of TUBβIII was found in the nucleus, in this case the treatment with PTX did not further modify its expression. In this experimental context, Ramucirumab treatment resulted in a significant decrease in the nuclear levels of the protein, in contrast to the combined treatment of PTX with Elacridar which induced a further significant increase in expression levels of TUBβIII. In conclusion, among the three drugs analyzed, only Ramucirumab treatment caused the reduction of TUBβIII protein in PTX-resistant cells (**Figure 3**, panel C).

### 3.4 Ramucirumab and Elacridar restore the pro-apoptotic action of Paclitaxel in resistant GC cells

The effects of Ramucirumab and Elacridar on PTX-mediated apoptosis were investigated in both PTX sensitive end resistant cell lines. The cells were subjected to single or combined drug treatments and 48 hsr later the effects on apoptosis were evaluated by using Annexin V cytofluorimetric assay. In HGC27-S cells, PTX or Ramucirumab alone caused a slight increase in percentage of total apoptotic cells, however, the combined treatment of the two drugs boosted this increase significantly compared to the percentage of apoptosis found in the control ones (29% *Vs.* 19.6% of total apoptotic cells). In the KATOIII-S both PTX and Ramucirumab exerted the same pro-apoptotic action compared to the percentage of apoptosis found in the control ones (36.7% *Vs.* 26.5% of total apoptotic cells), once again their combination significantly enhanced this effect compared to single drug treatment (44% *Vs.* 36.7% [PTX] and *Vs.* 26.5% [Ramucirumab]). In both sensitive lines the combination of PTX or PTX-Ram with Elacridar did not increase the percentage of apoptotic cells. On the other hand, in both resistant lines, while the Ramucirumab retained its pro-apoptotic activity when administrated both alone (29.2% *Vs.* 22% in HGC27-R and 37% *Vs.* 28% in KATOIII-R) or in combination with PTX (33.2% *Vs.* 22% in HGC27-R and 43.5% *Vs.* 28% in KATOIII-R), the pro-apoptotic action of the PTX was re-established only in presence of the Elacridar as documented by an increase of apoptotic cells, reaching the 40.5% and 37% when these two drugs are concomitantly administrated in HCG27-R and KATOIII-R respectively. Further increase was obtained combining Elacridar with Ramucirumab and PTX in both resistant lines (46.5% in HCG27-R and 48% in KATOIII-R). A representative apoptosis profile for HCG27 cells and the corresponding graphs including that of KATOIII cell line were reported respectively in panels A and B of **Figure 4**. Furthermore, the expression of proteins actively involved in inducing apoptosis were investigated both in HGC27-S/R and KATOIII-S/R cells and the results were shown in the panel C of **Figure 4**. In both HCG27 and KATOIII PTX-sensitive cells, PTX (4 nM for HGC27-S and 2 nM for KATOIII-S cells) caused a strong activation of the pro-apoptotic cascade JNK/JUN as well as the activation of both caspase 3 and 7 revealed in their cleaved active form. The combined treatment of Ramucirumab and PTX determined a further increase of these pro-apoptotic markers. In this context, the addition of Elacridar to the combined treatment of Ramucirumab and PTX did not lead to a further increase in the expression levels of apoptotic markers. In both PTX-resistant cells, where the levels of the markers analyzed were comparable in untreated cells and those treated with PTX alone, treatment with Ramucirumab not only induced the expression of pro-apoptotic factors, but also had a synergistic action in combination treatment with PTX. In this case, Elacridar also proved effective in combination with PTX in restoring the inductive effect which was furtherly enhanced in the triple treatment PTX-Elacridar-Ramucirumab (**Figure 4**, panel C).

### 3.5 Ramucirumab and Elacridar restore the inhibitory effects of Paclitaxel on cell migration

**Figure 5:**
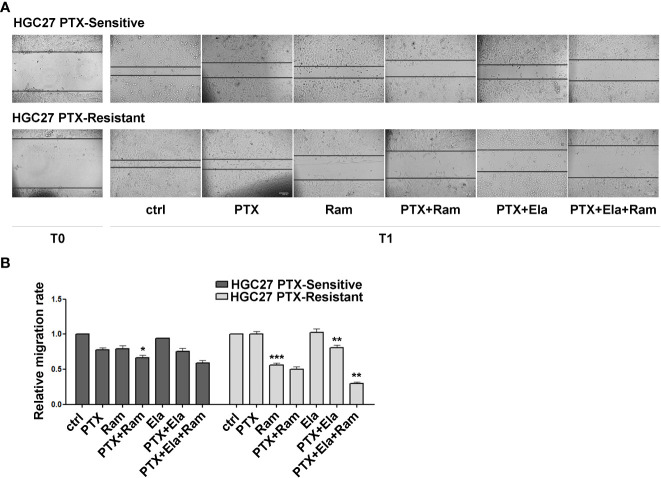
The effects of both Ramucirumab and Elacridar on PTX-mediated inhibition of cell migration. **(A)** scratch assay assessed on HGC27-S and HGC27-R treated with 4 nM PTX, 100 μg/mL Ram and 100 nM Ela alone or in combination. The cells were microscopically analyzed at the time of the scratch (T0) and after 24 hrs (T1); **(B)** The relative migration rate was calculated by setting the percentage of migration of the control cells at time T1 equal to 1 and comparing the percentage of migration of the cells after each drug treatment to this value. The experiments were performed in triplicate and the mean values ± SD were plotted in the relative graph. Statistical analysis was assessed comparing the values obtained using single drug treatment to those of corresponding untreated cells and the combined treatments to those of the single treatments, *p < 0.05; **p < 0.01; ***p < 0.001.

The effects of Ramucirumab and Elacridar in combination with PTX have also been investigated on the cellular motility of HGC27 sensitive or resistant to PTX. In this regard, a scratch assay was performed on both cell lines after single or combined treatments with the drugs. The results shown in **Figure 5** revealed that in HGC27-S cells both PTX and Ramucirumab reduced the migration rate by 22% (0.78 *Vs.* 1) and 21% (0.79 *Vs.* 1) respectively after 24 hrs (T1) of treatment. However, the reduction was grater when these two drugs were administrated simultaneously (0.67 *Vs.* 1). The addition of Elacridar to PTX or the PTX- Ramucirumab combination had no significant effect in further inhibition of cell migration. Again, in HGC27-R cells, as expected, PTX alone had no effect. A significant inhibition was observed after treatment with Ramucirumab alone (0.44 *Vs.* 1), while the PTX- Ramucirumab combination produced no further noteworthy effects with respect to treatment with Ramucirumab alone (0.5 *Vs.* 1). Elacridar in combination with PTX resulted in a significant reduction in migration (0.81 *Vs.* 1) which was even more pronounced after simultaneous treatment with the three drugs (0.3 *Vs.* 1) (**Figure 5**, panels A and B).

### 3.6 Role of PTX and Ramucirumab in VEGFA/VEGFC secretion in both sensitive and resistant GC cell lines

**Figure 6:**
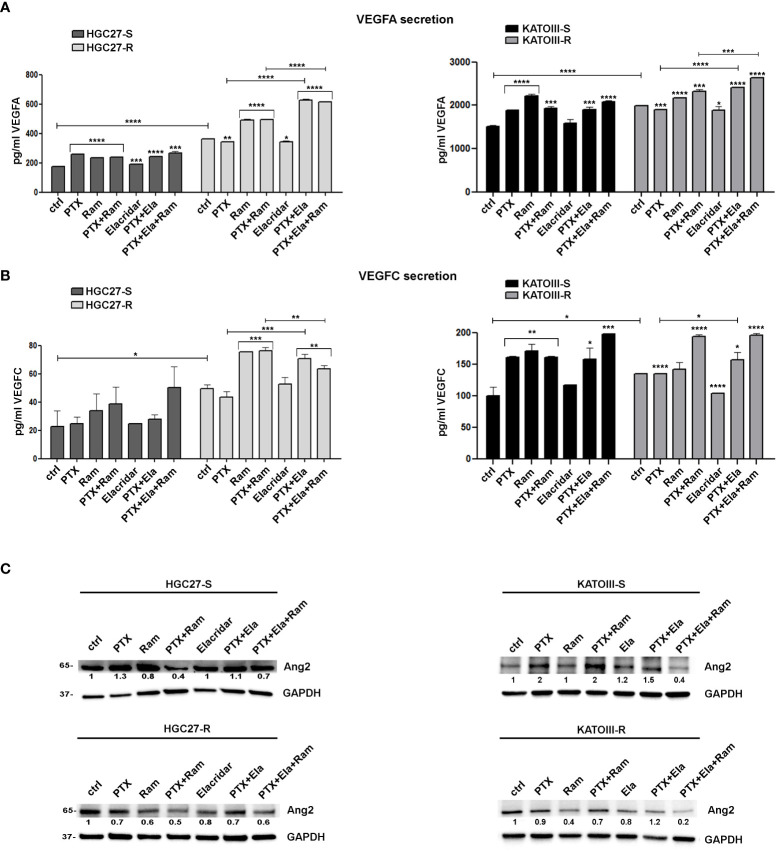
The effects of PTX and Ramucirumab on VEGFA/VEGFC secretion. The ELISA assays were assessed on HGC27-S/R and KATOIII-S/R treated with 4 nM PTX (HGC27) or 2 nM PTX (KATOIII), 100 μg/mL Ramucirumab (Ram) and 100 nM Elacridar (Ela) alone or in combination. The concentration of VEGFA **(A)** or VEGFC **(B)** was determined in the medium and normalized for the cell number. The values ± SD, obtained from three independent experiments expressed as pg/mL were shown in the relative graphs. Statistical analysis was assessed comparing the values obtained using single drug treatment to those of corresponding untreated cells and the combined treatments to those of the single treatments. The values of untreated PTX-resistant cells were compared with those of untreated PTX-sensitive ones, *p < 0.05; **p < 0.01; ***p < 0.001; ****p < 0.0001. **(C)** Representative Western Blotting analysis performed in HGC27-S/R and KATOIII-S/R cells relative to the expression of Ang2. Nuclear protein extracts have been normalized using stain free technology. GAPDH has been used as a normalizer of cytoplasmic protein extracts. Values indicating normalized expression levels are shown on each band.

VEGFA/VEGFC secretion experiments in the culture medium were conducted in both cell lines HGC27-S/R and KATOIII-S/R treated with PTX (4 nM for HGC-27-S/R and 2 nM for KATOIII-S/R cells), 100 μg/mL Ramucirumab and 100 nM Elacridar. The conditioned media were collected after 48 hrs of single or combined drug treatments and the concentrations of the two growth factors were detected using specific ELISA kits, as described in the Method section. The results obtained were reported in **Figure 6**. In HGC27-S both PTX and Ramucirumab led to an increase in the secretion levels of VEGFA (46% and 34% respectively) and no further increase was observed after the combined treatment, as well as the combined treatment of PTX and Elacridar did not increase secretion compared to PTX alone. In HGC27-R cells, the level of secreted VEGFA was significantly higher (106%) than in sensitive cells and was not modified by PTX alone. However, both Ramucirumab alone and combined treatment with PTX and Elacridar resulted in significant increases in secreted protein (35.3% and 72.6% respectively). VEGFA secretion levels in KATOIII were eight-fold higher than in HGC27. In these cells, significant increase in secretion upon treatment with both PTX (24.7%) and Ramucirumab (46.5%) was found and again without further increases after combined PTX- Ramucirumab treatment. A slight increase was detectable after treatment combined with PTX-Ela (1%), while the triple treatment PTX-Ela-Ram was ineffective compared to the treatment with PTX-Ramucirumab combination. In KATOIII-R there was an increase in VEGFA secretion (31.4%) compared to KATOIII-S. Even in this resistant cell line, PTX did not produce effects unlike Ramucirumab, which once again led to an increase in protein in the medium both alone (9.1%) and in combination with PTX (16.7%) compared to untreated cells. The combined treatment PTX-Elacridar induced further secretion of VEGFA (27.1%) compared to single treatment with PTX or Elacridar. The triple combination treatment PTX-Elacridar-Ramucirumab induced a further secretion of VEGFA (13.8%) compared to the dual combination treatment PTX-Ramucirumab (**Figure 6**, panel A).

The results obtained by analyzing the secretion of the other ligand of VEGFR2, VEGFC, in both cell lines were in line with those obtained for VEGFA confirming both the greater affinity of Ramucirumab to bind VEGFR2 compared to VEGFA and VEGFC ligands and the action of PTX in stimulating the secretion of pro-angiogenic factors, this last action was restored by Elacridar in PTX-resistant cells (**Figure 6**, panel B).

By extending the analysis to the expression levels of Ang2 in sensitive and resistant cells in the same experimental conditions, the results showed that PTX treatment caused induction in Ang2 levels in sensitive cells while it was ineffective in the resistant one, but its effect was restored in combination with Elacridar. Whereas treatment with Ramucirumab resulted in a slight decrease in Ang2 levels in PTX-sensitive cells and a marked decrease in resistant cells, this effect was pronounced after PTX-Ram combination. The triple combination PTX-Ela-Ram led to a slight increase in protein expression compared to the combination PTX-Ram in HGC27 cells, whereas a further decrease was observed in KATOIII cells (**Figure 6**, panel C).

### 3.7 Exosome release as a mechanism for spreading PTX resistance

#### 3.7.1 Exosomes characterization

**Figure 7:**
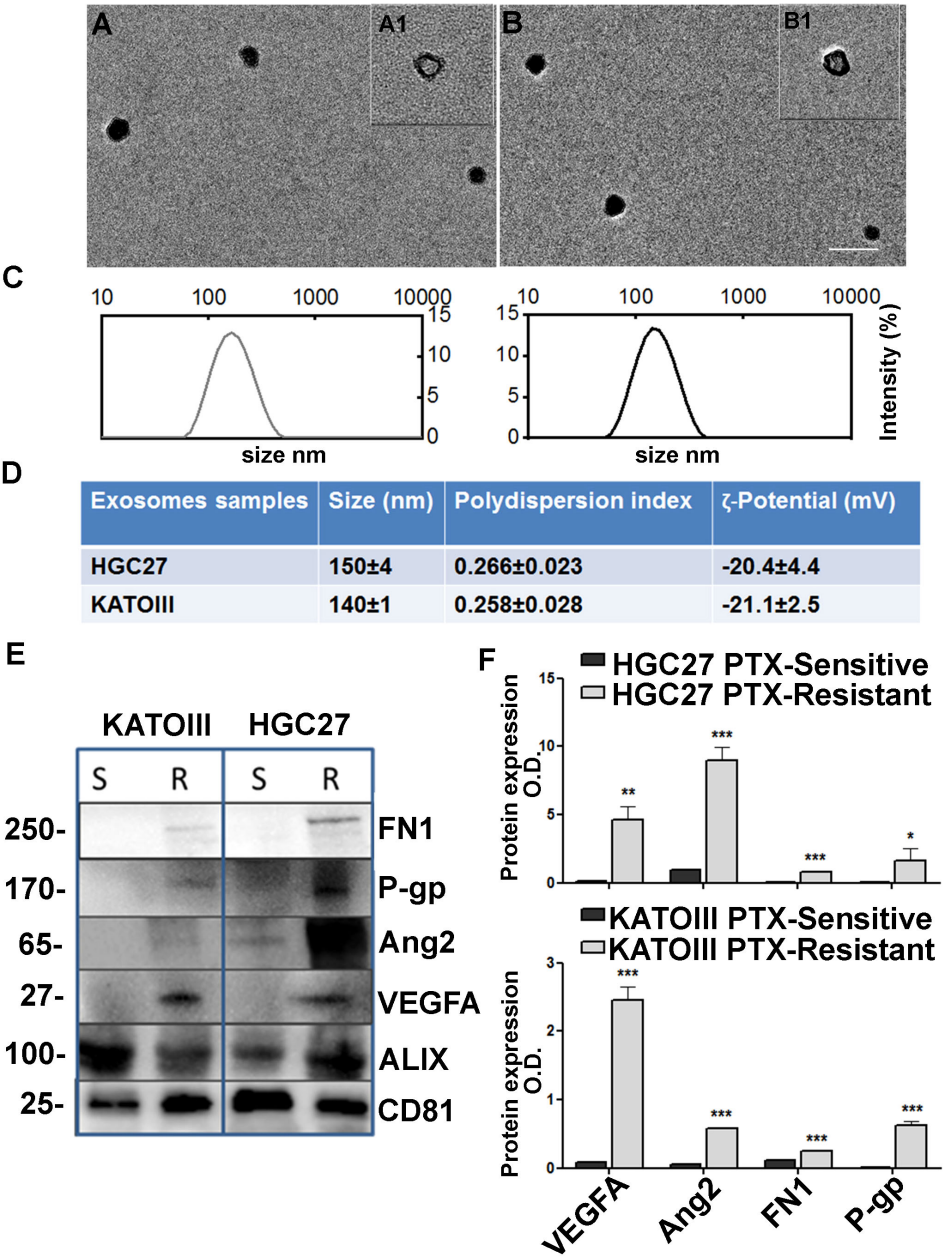
Exosome characterization. TEM micrographs (scale bar 200 nm) of exosomes extracted from culture medium of KATOIII cells **(A)** and HCG27 **(B)** at two different staining times 30 s (**A1**, **B1**) and 60 s (**A**, **(B)**; **(C)** DLS-intensity distribution of exosomes derived from KATOIII and HGC27 respectively; **(D)** Table reporting the data obtained by DLS analysis and ζ-potential measurements (Mean ± SD, n = 3); **(E)** The representative WB for exosomes cargoes proteins extracted from KATOIII and HGC27 both sensitive and resistant to the PTX, namely: FN1, P-gp, Ang2, VEGFA and housekeeping proteins for exosomes namely ALIX and CD81; **(F)** The graphs represented the WB bands densitometry normalized for CD81 for HGC27 (upper graph) and KATOIII. For each protein, statistical analysis was assessed comparing the normalized value of PTX-resistant cells to that of sensitive ones, ***p<0.001; **p<0.01; *p<0.05.

Spherical vesicles, characterized by a bilayer structure and size ≤ 150 nm, can be observed in the TEM micrographs the exosomes isolated from culture medium of HGC27 and KATOIII cells (**Figure 7**, panels A, A1, B and B1). The size values obtained by the DLS analysis were ≤ 150 nm for all the investigated samples, thus in perfect accordance with the TEM observation (**Figure 7**, panels C, D). As expected, the ζ-potential measurements confirmed the presence of a negative charge onto the exosomes surface, composed of phospholipid-based cell membrane (**Figure 7**, panel D).

Western Blotting analysis was performed to evaluate the potential capability of exosomes to vehiculate proteins such as FN1, P-gp, Ang2, and VEGFA, and the difference between EXO-R and EXO-S. In **Figure 7**, panel E the representative WB for exosomes cargoes proteins extracted from HGC27 and KATOIII both sensible and resistant to the paclitaxel were reported. Housekeeping proteins for exosomes named ALIX and CD81 have been used to normalize the expression levels of the investigated proteins. As shown, the difference between EXO-S and EXO-R in both cell lines was substantial.

Exosomes derived from both HGC27-R and KATOIII-R showed an over-expression of all cargo proteins compared to exosomes derived from PTC-sensitive cells. The graphs reported in panel F represented the WB band densitometry normalized for CD81.

#### 3.7.2 Exosomes derived from PTX-resistant cells as active messengers of resistance characteristics

**Figure 8:**
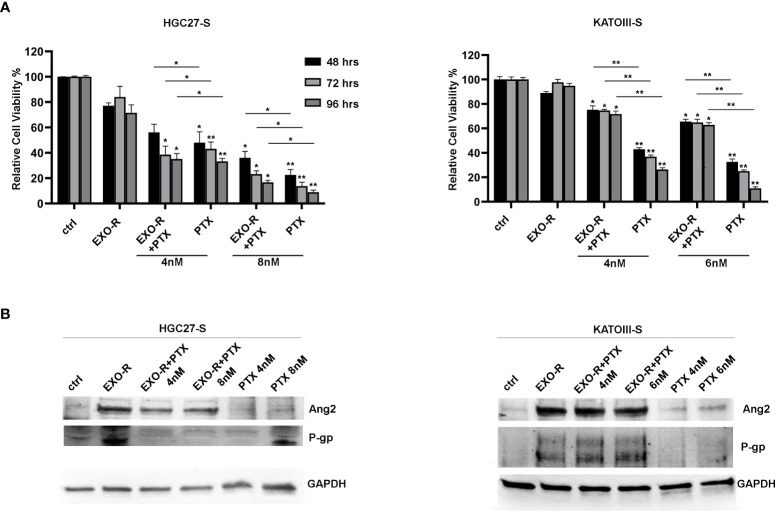
Effects of exosomes derived from PTX-resistant cells on PTX-sensitive GC cells. **(A)** MTS assay performed on HGC27-S and KATOIII-S cell lines treated with exosomes (EXO-R) extracted by HGC27-R and KATOIII-R cells medium respectively. Different PTX concentrations (4 nM and 8 nM for HGC27 and 4 nM and 6 nM for KATOIII) were added for 48, 72, 96 hrs. The percentages of cell viability have been compared to that of untreated cells (100%). Significance was also calculated between each sample treated with PTX alone and the respective combined PTX+EXO-R treatment. The experiments were performed in triplicate and the mean values ± SD were plotted in the relative graph. *p < 0.05; **p < 0.01; **(B)** Expression analysis of P-gp and Ang2 proteins in PTX-sensitive GC cells treated with/without EXO-R and with/without PTX as described above.

Cell viability experiments were performed treating HGC27-S and KATOIII-S cell lines with or without exosomes extracted from HGC27-R and KATOIII-R cells respectively. In addition, the cells were treated with or without different PTX concentrations for 48, 72, 96 hrs. The results of the MTS experiments have been reported in **Figure 8** where all the percentages of cell viability have been compared to that of untreated cells (100%). In HGC27-S, by comparing the growth of co-treated cells with EXO-R and PTX to that after treatment with only PTX, it was found that treatment with EXO-R significantly reduced the inhibitory action of 8 nM PTX already after 48 hours of incubation (48.01 ± 8.65 *Vs.* 22.5 ± 4.3%), this effect was further increased after 72 (43.17 ± 5.35 *Vs.* 13.72 ± 3.15 %) or 96 hrs (33.2 ± 2.365 *Vs.* 8.98 ± 1.65%), highlighting PTX de-sensitizing caused by drug-resistant cells derived exosomes. The lowest concentration of Paclitaxel (4 nM) has a significant effect on cell viability after 72 and 96 hrs, comparing it with cells treated simultaneously also with exosomes (respectively 38.51±6.68 *Vs.* 23,22 ±2.6 and 35.12 ± 4.3 *Vs.* 16.7 ± 1.65). In KATOIII-S cells both concentrations (4 and 6 nM) of PTX caused a significant reduction of cell viability (**p<0.001) that was restored combining PTX and exosomes derived from resistant cells medium (EXO-R). These effects were significant at both concentrations of PTX and for all incubation times analyzed. Specifically, treating the cells with 4 nM PTX for 48 hrs resulted in a live cell percentage of 42.9%, which increased to 75.2% with PTX EXO-R combined treatment. This effect was increasingly pronounced as incubation time and PTX concentrations used increased. The treatment of sensitive cells with EXO-R alone did not significantly change cell viability (**Figure 8**, panel A).

Furthermore, the expression analysis of some of the proteins involved in resistance mechanisms revealed that treating both HGC27-S and KATOIII-S whit EXO-R and the combination of EXO-R with PTX at different concentration for 96 hrs determined a significative increase in the levels of P-gp and Ang2 expression compared to the untreated control. Under these experimental conditions the effects of PTX alone as well as those of the combined PTX and EXO-R were not significant. Although there was a slight increase in the levels of the two proteins after single drug treatment (**Figure 8**, panel B).

## 4 Discussion

PTX is a known antimitotic agent, which blocks the disassembly of interphase microtubules causing cell cycle arrest (1). Paclitaxel, being an antimitotic agent, prevents cells from progressing beyond the G2 phase (29, 30), but its effect at other stages of the cell cycle cannot be ruled out. An inhibitory action during the S phase of the cell cycle in other cell models is also reported by other authors (2, 3). PTX was the first taxane discovered and proven to be effective in several cancers, including GC (6, 31). Taxanes are used in perioperative chemotherapy of resectable GC (Docetaxel) and in metastatic disease where PTX is administered both in the first and in second line therapy in combination with the anti-angiogenic antibody Ramucirumab (6, 7, 32). Although taxanes are a widely used and effective “weapon” in the therapy of cancer, resistance to these drugs and in particular to PTX is observed in a large percentage of cases (33). The mechanism of taxane-mediated resistance is a process that involves several intracellular signal cascades and it has not been still fully understood.

Combined therapies represent a valid option to evade the resistance mechanisms thus increasing the effectiveness of therapy in terms of progression free survival (PFS) and overall survival (OS) (7, 11, 13). Ramucirumab is used both as monotherapy and in association with PTX for advanced GC in patients with disease progression after platinum and fluoropyrimidine therapy (7, 8). Ramucirumab binds VEGFR2 about eight times more efficiently than VEGFA (10, 34, 35), thereby targeting not only neo-angiogenesis in endothelial cells (9), but also the growth and motility of epithelial cancer cells through the support of the autocrine cycle. The combined regimen with Ramucirumab increases the effectiveness of PTX and could prevent the resistance to this drug even in patients receiving first-line taxane-based chemotherapy (13). A previous study provided clear evidence of the synergistic effect of PTX and Ramucirumab combination in inhibiting the growth and migration of cells from different human GC lines (23). The data stressed the importance of the combined treatment in order to strongly inhibit all the main molecules of the signaling pathways targeted by the two drugs, thus preventing possible reactivations due to cross-talk phenomena.

The present study focused on the analysis of molecular processes contributing resistance in GC lines with PTX-induced resistance. The availability of two independent GC lines, HGC27-R and KATOIII-R, proved to be a crucial tool to investigate some of the mechanisms involved in resistance that was induced by continuous treatment with small concentrations of PTX. Differentially expressed genes in PTX-resistant cell lines versus sensitive ones were identified within a pre-established panel of 88 genes involved in angiogenesis. The complex gene network was highlighted with the STRING software, which emphasized the key role of VEGF/VEGFR family members. Among the overexpressed genes, the ligands VEGFA and VEGFC, the VEGFR3 receptor or closely related genes, such as Ang1/2, were identified. Among down-regulated genes, KDR, the gene coding for VEGFR2, was identified. Real-time PCR experiments with specific primers confirmed these results for the KDR, VEGFA, VEGFC and ANGPT2 genes. The results were validated for some of the genes investigated in both PTX-sensitive and resistant cell lines after single or combined treatment with PTX and Ramucirumab. In the sensitive lines, as expected, PTX treatment led to a decrease in VEGFR2 and an increase in VEGFA, VEGFC and Ang2, whereas Ramucirumab led to a decrease in the expression levels of all genes investigated. The combination treatment did not lead to a further significant decrease in VEGFR2. Combined PTX and Ramucirumab treatment, due to the opposite action of the two drugs, resulted in an increase in the expression of VEGFA, VEGFC and Ang2 markers compared to the treatment with Ramucirumab alone, but still a decrease in the same proteins, compared to untreated cells. The resistant lines, characterized by an overexpression of the investigated growth factors, resulted more sensitive to the action of Ramucirumab than their sensitive counterpart. Thus, in these cells, while treatment with PTX was ineffective in changing the expression levels of the markers under investigation, treatment with Ramucirumab produced a significant effect in reducing their expression levels. The combination treatment resulted in a further reduction in the expression levels of VEGFR2 and Ang2, but not in VEGFA and VEGFC. It is widely accepted that the activation of VEGF/VEGFR2 signaling correlates with the overexpression of Ang2 by endothelial cells in the stroma surrounding the tumor. As a result, Ramucirumab could inhibit its expression both in endothelial and tumor cells. Therefore, VEGF and Ang2 can be considered two key factors of angiogenic switch that cooperate in vessel remodeling and in the formation of new structurally disorganized tumor vessels (36, 37).

As well recognized, the resistance mechanism induced by PTX was demonstrated to be multi-factorial (33). One of the factors involved in the decreased efficacy of PTX is the overexpression of membrane proteins responsible for the outflow of the taxane from the cell, which includes P-gp. These proteins belong to the super family of ABC conveyors and their inhibition can partially restore PTX activity in PTX-resistant cells (14–18). In both resistant cell lines used in this study an overexpression of P-gp was found and its antagonist of choice, Elacridar was used to inhibit the action of P-gp. The effect of combined Elacridar-PTX treatment, in restoring sensitivity and thus producing its cytotoxicity, was investigated (19, 20). The treatment with Elacridar of both resistant cell lines resulted in increased levels of PTX within resistant cells. In addition, the combined treatment PTX-Elacridar partially restored the inhibitory action of PTX in the process of cell growth.

The combined treatment of Elacridar and PTX restored the antimitotic action of PTX in both resistant lines. The results of the cell cycle analysis showed an increase in the percentage of cells in G2/M and a decrease of cells in G0/G1 after treatment with PTX in lines sensitive to its action, in the latter the combination with Elacridar slightly increases this effect. In resistant cells, however, this effect was observed only after combined treatment of PTX with Elacridar, given the increased presence of P-gp channels in these cells. These results have also been confirmed by a cell cycle analysis that allows the distinction of the different phases of the cycle passed by the cells in culture thanks to a different color. Furthermore, indirect effects of Ramucirumab on the cell cycle have been investigated through modulation of some of the key proteins actively involved in cell cycle progression and thus in the proliferative process. The combined treatment of Ramucirumab and PTX resulted in a significant reduction in the levels of nuclear expression levels of β-catenin, NFkB and cyclin B1 (cycB1), in both sensitive and resistant cells. Dominant effects of PTX and Ramucirumab combination were in line with data from dose-response experiments, reported in previous study (23). The results showed that 100 µg/mL Ramucirumab enhanced the inhibition exerted by PTX concentrations close to its IC50. According to the Chou and Talalay method, the combination indices (CI) calculated for these drug combinations were ≤1, demonstrating that the drugs exerted their effect synergistically in both the HGC27 and KATOIII PTX-sensitive cell lines. The simultaneous treatment with PTX, Elacridar and Ramucirumab caused a further decrease of the abovementioned proteins, in PTX-resistant cells. β-catenin and NFkB are both involved in the regulatory mechanism of VEGFA expression (38, 39). CycB1 is a transcription factor examined due to its key role in the transition from G2 phase to mitosis and its role in the mechanism of taxane resistance (40).

Moreover, PTX treatment determined nuclear accumulation of TUBβIII, a specific isoform of tubulin, which makes the microtubules’ formation process more dynamic (4, 41). PTX-resistant cells showed a nuclear accumulation of TUBβIII, whose levels decreased significantly after treatment with Ramucirumab (13). The anti-angiogenic drug, not only blocks the VEGF/VEGFR pathway, but also had an important effect on reduction of nuclear TUBβIII, thereby partially restoring the antimitotic effect of PTX. Therefore, PTX-Ramucirumab treatment had a synergistic effect in blocking resistant cells growth (23). In resistant cells, the overall effect of the PTX-Elacridar combined treatment was inhibitory of tumor growth, nevertheless these combined drugs determined a nuclear accumulation of TUBβIII, as well as the induction of pro-angiogenic genes (VEGFA, VEGFC, Ang2).

Analysis of the apoptotic process in PTX-sensitive cells showed that a significant pro-apoptotic action resulted from the combination of PTX and Ramucirumab rather than from treatment with the individual drugs, and this again was in line with the synergism resulting from their combination (23). The effects of Elacridar and Ramucirumab on increasing sensibility of PTX resistant lines, have been significantly appreciated in the induction of the apoptotic process, which became significant after combined treatments with PTX-Ramucirumab and PTX-Elacridar and even more evident after triple PTX-Ela-Ram treatment. The pro-apoptotic cascade JNK/JUN as well as the activation of both cleaved active forms of caspase 3 and 7 were strongly induced after the combined treatment described above. By restoring PTX activity with Elacridar and blocking VEGF/VEGFR signaling with Ramucirumab in HGC27-R, there was also a significant reduction in the rate of cell migration which was reduced by 70% after triple drug treatment.

The increased expression of VEGFA and other pro-angiogenic factors in tumor cells in patients with GC is considered a negative prognostic factor (42) and is associated with an immunosuppressive tumor microenvironment (43). In contrast, its predictive role in response to anti-angiogenic therapy is still debated. In an undergoing observational study, the authors are evaluating the correlation between VEGFA amplification and response to Ramucirumab-PTX second-line therapy in patients with GC (44). Regarding the serum levels of VEGFA, previous analyses revealed an increase of circulating VEGFA due to Ramucirumab-PTX therapy as a result of the competition between Ramucirumab and VEGFA to the binding site of VEGFR2 (45). Ramucirumab’s binding affinity to VEGFR2 is greater comparing to VEGFA/VEGFC ligands, therefore during therapy the antiangiogenic binds VEGFR2 by displacing its ligands. Previous studies in patients with metastatic GC undergoing second-line therapy with Ramucirumab and PTX, revealed that the increase in serum level of circulating VEGFA, as well as a decrease in circulating levels of Ang2 indicated the effectiveness of therapy (46). The result of the present *in vitro* study confirmed an increase in levels of pro-angiogenic factors secreted in the culture medium following treatment with Ramucirumab. Moreover, PTX and PTX-Elacridar caused an increase in expression levels of the same growth factors in sensitive and resistant cells respectively. Therefore, after treatments with both Ramucirumab and PTX, an increase in secretion levels of VEGFA and VEGFC in these cell lines was observed, which was even more evident after combined treatments with PTX/Ram and PTX/Ela/Ram. These effects were still more significant in resistant lines (particularly in KATOIII-R), which were characterized by an overexpression of the investigated factors but also by increased sensitivity to the inhibitory effect of Ramucirumab. In addition, the analysis of Ang2 expression levels in the same cells confirmed its decrease after treatment with Ramucirumab and its increase after PTX treatment in sensitive cells and after PTX-Elacridar combination in resistant cell lines. In PTX-sensitive cells, combined treatment with PTX and Ramucirumab caused an increase in Ang2 levels compared to treatment with Ramucirumab only but were still lower than those of control cells or treated with PTX. The same effect was found in PTX-resistant cells after combined treatment with PTX, Elacridar and Ramucirumab.

Finally, many of the features that confer resistance to drug treatment can be spread in the tumor microenvironment through exosomes (21, 22). Therefore, this kind of extracellular communication plays a key role in cancer progression, invasion, neovascularization, metastasis, and drug resistance. In several tumors, it has been proven that ABC conveyors (47) as well as growth factors (48) can also be carried through these vesicles. Exosomes released by cancer cells transfer oncogenic molecules not only to other malignant cells, but also to stromal cells, and can determine the malignant transformation of normal cells (21, 22). The presented *in vitro* experiments have shown that, also in GC cells, exosomes derived from PTX-resistant cells, compared to those derived from sensitive cells, overexpress VEGFA, Ang2, FN1 and P-gp. Moreover, PTX-sensitive cells have been shown to acquire some of the typical characteristics of drug resistance cells when they were treated with supernatant of PTX-resistant cells. These data confirmed that exosomes act as efficient messengers in intercellular communication, and that their active release from drug-resistant cells in their surroundings spreads the resistance to sensitive ones (49). The mechanism of transfer of P-gp *via* exosomes known as horizontal transfer, has been already described as a mechanism in breast cancer (50) determines the spreading of resistance within a tumor mass, following the detachment from resistant tumors of vesicles rich of P-gp, assisted by other interacting proteins that facilitate the process (51). P-gp has been already detected in exosomes from gastric cell lines resistant to 5-fluorouracile, cisplatin and vincristine (52, 53). Blocking the release of exosomes by GW4896 reversed drug resistance or increasing the release of exosomes carrying mir-107, which repressed the high mobility group A2/mTOR/P-gp transcriptional axis (52), suggesting that the transfer of P-gp *via* exosomes from HGC27-R and KATOIII-R is likely part of a resistance mechanism of these cells. This was the first study describing the transfer of P-gp through exosomes in PTX-resistant gastric cell lines. Furthermore, our data are in line with previous observations showing that exosomal P-gp in the serum of prostate cancer patients is indicative of taxane resistance (54).

## 5 Conclusions

Paclitaxel resistance involves several processes, many of which are not yet fully understood. The experiments reported in the present study explored some of the hallmark of PTX-mediated resistance through the comparative analysis of two human GC cell lines with taxane induced resistance and their respective sensitive counterparts. A relevant feature was the overexpression of factors such as VEGFA, VEGFC and Ang2, which are not only pro-angiogenic, but also support the growth of tumor cells. A second significant characteristic was the induction of TUBβIII, an isoform of tubulin that opposes microtubule stabilization, in both resistant lines. A third key element was the finding of overexpression of P-gp one of the MDR transporters responsible for the efflux of chemotherapy from the cells. Therefore, these elements account for the increased sensitivity to both Ramucirumab and Elacridar found in PTX-resistant cells compared to drug-sensitive cells. The first significantly reduced the expression not only of angiogenic molecules but also of TUBβIII, the second restored the access chemotherapy, recovering its cytotoxicity due to anti-mitotic and pro-apoptotic effects. Finally, the results of this study highlighted the role played by exosomes in the transport and diffusion into the tumor microenvironment of some of the factors overexpressed in resistant cells, such as VEGFA, VEGFC, Ang2 and P-gp. Overall, these findings revealed the effectiveness of the combined triple treatment with PTX, Elacridar and Ramucirumab in bypassing PTX-induced resistance.

## Data availability statement

The datasets presented in this study can be found in online repositories. The names of the repository/repositories and accession number(s) can be found in the article/**Supplementary Material**.

## Ethics statement

This research was approved by scientific and technical committee of IRCCS “S. De Bellis” and funded by Italian Ministry of Public Health (n.02/RC2021); “Italian Association for Cancer Research” (AIRC IG21408) and PON BIO-D project: “Development of Diagnostic Biomarkers for precision medicine and personalized therapy” (ARS01_00876).

## Author contributions

Conceptualization: RD’A, CM, CL, GG, and MR; methodology: RD’A, AS, MC, CR, GL, MS, MP, and ND; software: AS, GL, and FR; validation: MC, CR, MS, MP, CM, and RD’A; formal analysis: AS, LC, MM, JK, and FR; investigation: AS, MC, MR, and RD’A; resources: MC, CR, MS, MP, ND, and RD’A; data curation: AS, LC, JK, MR, MM, and RD’A; writing-original draft preparation: AS, MC, CR, MS, MP, ND, and RD; writing-review and editing: AS, MC, CR, MS, MP, MM, JK, MR, CL, GG, CM, and RD’A; visualization: CL, GG, CM, and RD’A; supervision: CL, GG, CM, and RD’A; project administration: CM and RD’A; funding acquisition: CR, MS, GG, and CM. All authors contributed to the article and approved the submitted version.
