# Supplementary material for: The multiple combination of Paclitaxel, Ramucirumab and Elacridar reverses the paclitaxel-mediated resistance in gastric cancer cell lines
Source: Front Oncol. 2023 Feb 16;13:1129832. doi: 10.3389/fonc.2023.1129832 (PMC9978398; doi:10.3389/fonc.2023.1129832)
Supplement: Supplementary file 1 [file DataSheet_1.pdf]

Supplementary Material

The multiple combination of Paclitaxel, Ramucirumab and Elacridar reverses the Paclitaxel-mediated resistance in Gastric Cancer cell lines

Annalisa Schirizzi[1], Marialessandra Contino[2], Livianna Carrieri[3], Chiara Riganti[4], Giampiero De Leonardis[1], Maria Principia Scavo[3], Maria Grazia Perrone[2], Morena Miciaccia[2], Joanna Kopecka[4], Maria Grazia Refolo[1], Claudio Lotesoriere[5], Nicoletta Depalo[6], Federica Rizzi[6,7], Gianluigi Giannelli[8], Caterina Messa[1*] and Rosalba D'Alessandro[1*]

*** Correspondence:**

Caterina Messa: caterina.messa@irccsdebellis.it; Phone number: +39 0804994178

Rosalba D'Alessandro: rosalba.dalessandro@irccsdebellis.it; Phone number: +39 0804994178

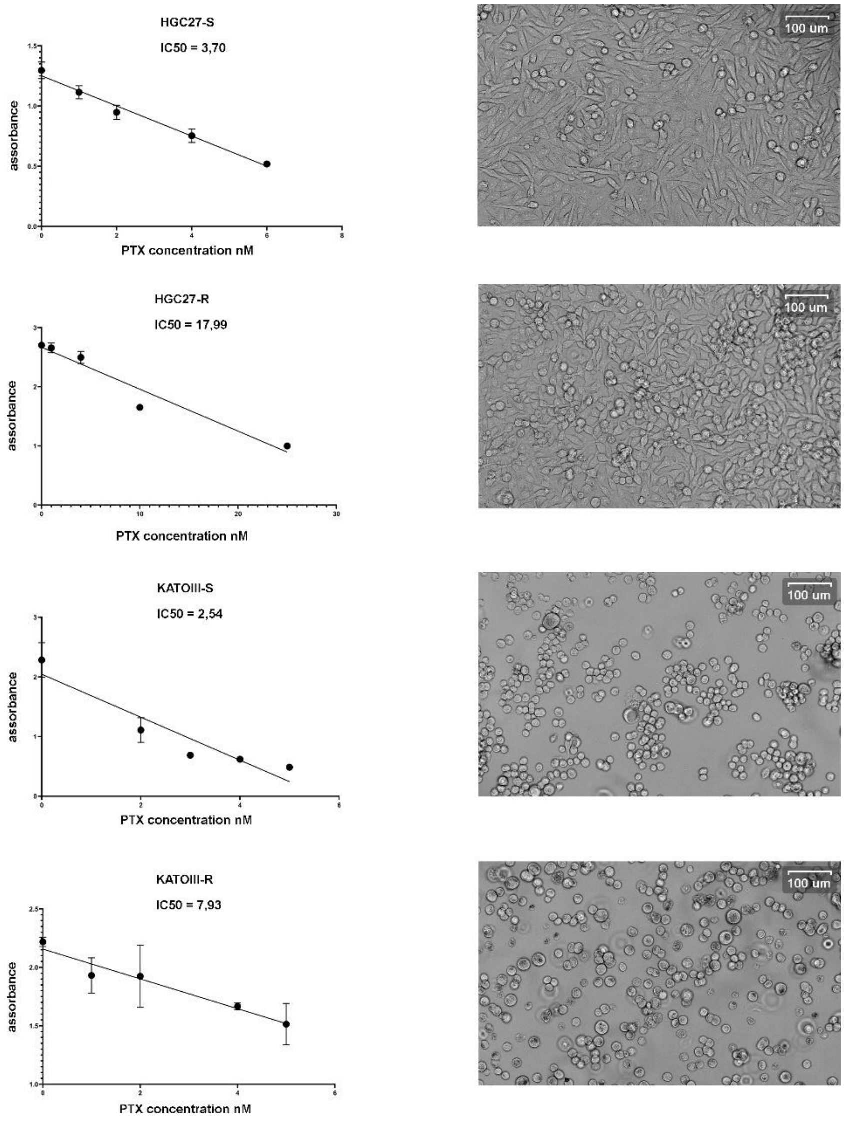

Supplementary Figure 1. Cell morphology and half-maximum inhibitory concentration of PTX in HGC27-S/R and KATOIII-S/R.

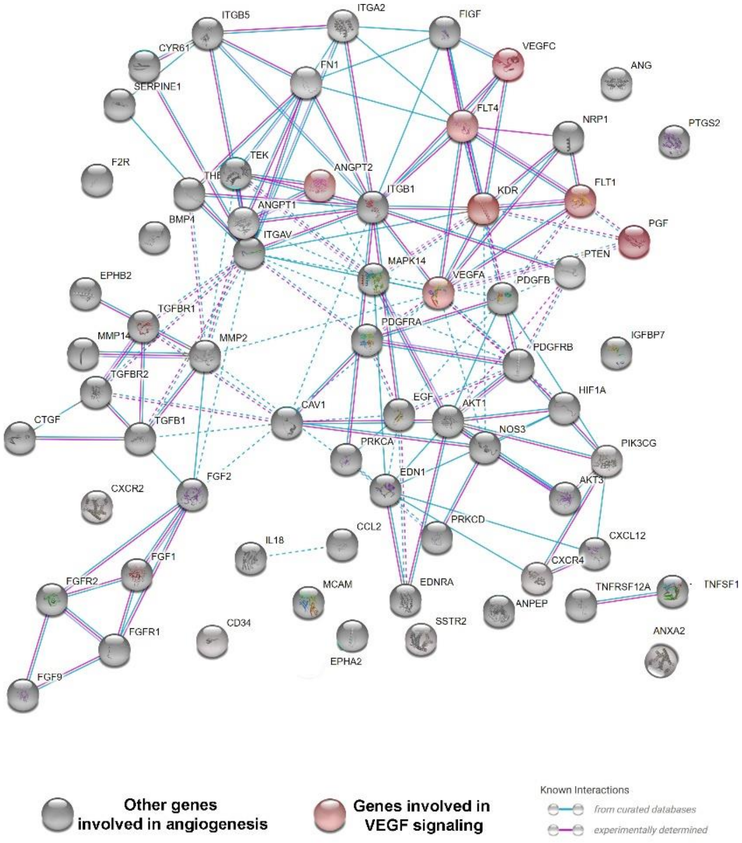

Supplementary Figure 2. The network gene interconnections from data base (blue lines) or experimental obtained (red lines) with STRING program, in red color genes involved in VEGF signaling.

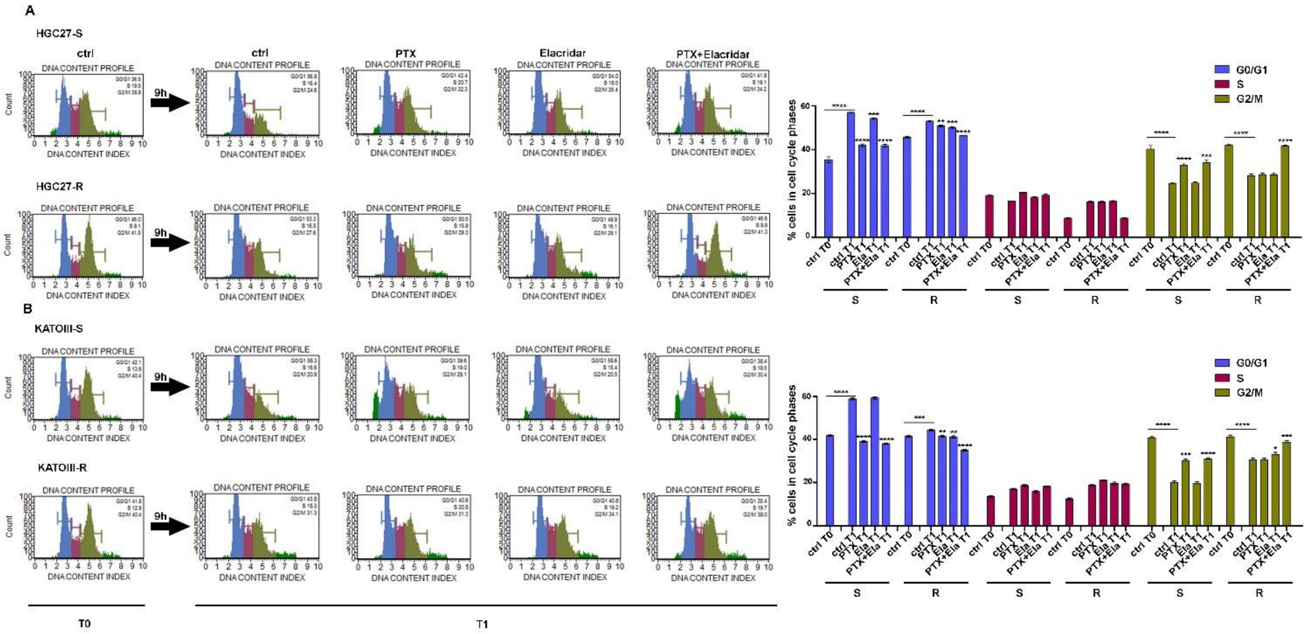

Supplementary Figure 3. HGC27-S/R (**A**) and KATOIII-S/R (**B**) human GC cells were synchronized in the G2/M phase of the cell cycle using nocodazole (T0). Two nM (KATOIII) or 4 nM PTX (HGC27) and 100 nM Ela alone or in combination were administrated to a subgroup of cells processed after 9 hrs from block release (T1).

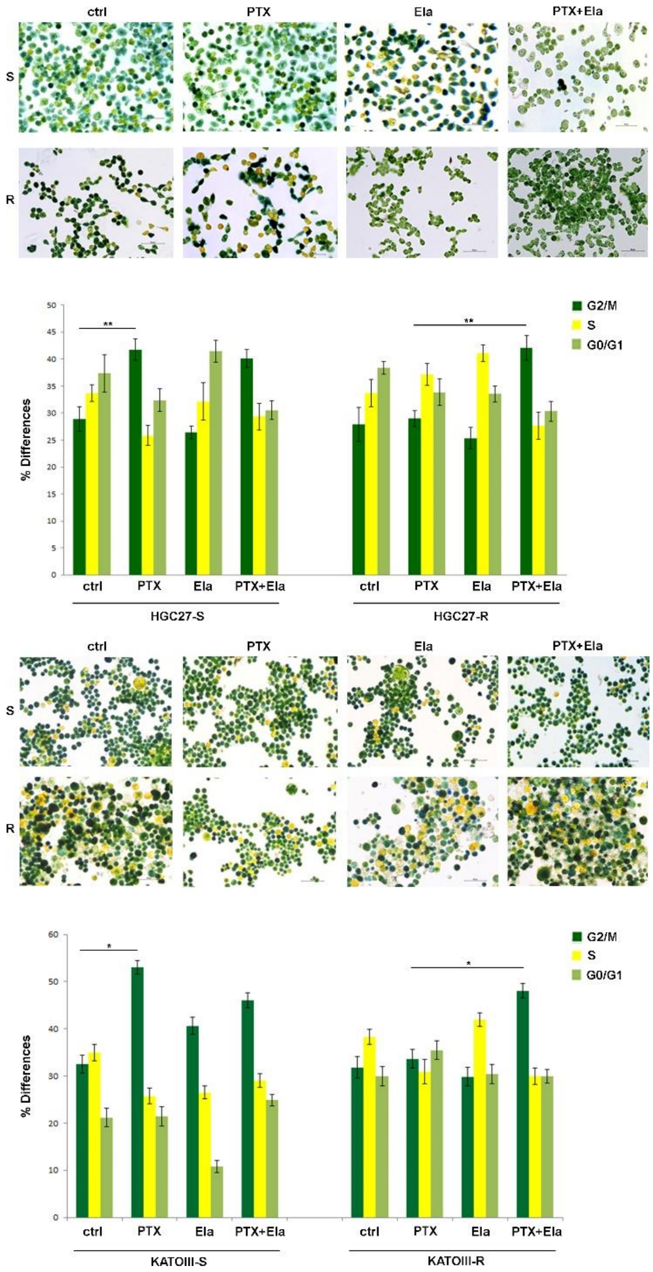

Supplementary Figure 4. Cell cycle progression assessed with the Cell-Clock™ Cell Cycle Assay in HGC27-S/R and KATOIII-S/R human GC cells treated with 2 nM (KATOIII) or 4 nM PTX (HGC27) and 100 nM Ela alone or in combination for 12 hrs.
